# The emerging importance of tackling sleep–diet interactions in lifestyle interventions for weight management

**DOI:** 10.1017/S000711452200160X

**Published:** 2022-08-14

**Authors:** Wendy L. Hall

**Affiliations:** Department of Nutritional Sciences, School of Life Course and Population Sciences, Faculty of Life Sciences and Medicine, King’s College London, London SE1 9NH, UK

**Keywords:** Diet, Sleep, Social jetlag, Adiposity, Weight loss

## Abstract

Sleep habits are directly related to risk of obesity, and this relationship may be partly mediated through food choices and eating behaviour. Short sleep duration, impaired sleep quality and suboptimal sleep timing are all implicated in weight gain and adverse cardiometabolic health, at least partly mediated through their associations with diet quality. Short-term sleep restriction leads to increased energy intake, and habitually short sleepers report dietary intakes that indicate a less healthy diet compared with adequate sleepers. Evidence is emerging that sleep extension interventions in short sleepers may reduce intake of sugars and overall energy intake. Poor sleep quality, night shift work patterns and social jetlag are also associated with lower diet quality and consumption of energy-dense foods. Incorporating sleep advice into weight management interventions may be more effective than energy-restricted diets and exercise advice alone. However, there are a lack of intervention studies that aim to lengthen sleep, improve sleep quality or adjust irregular sleep timing to investigate the impact on dietary intakes and eating behaviour in participants aiming to lose weight or maintain weight loss. Finally, future research should take account of individual characteristics such as age, sex, life stage and changing working practices when designing combined lifestyle interventions including sleep behaviour change for health and well-being.

The fundamental role of a healthy diet in preventing obesity and chronic diseases means that nutrition advice is an essential component of lifestyle interventions and dietary guidelines are a key public health policy tool. However, the most effective strategy to facilitate adherence to dietary guidelines and weight management programmes continues to be the subject of intense discussion^(1,2)^. At a population level, adherence to dietary guidelines is low. A combination of diet data from four major UK databases, including the National Diet and Nutrition Survey (2012–2017), indicated that 9–14 % of the population were adhering to ≤ 1 out of a total of nine Eatwell Guide dietary guidelines^(3)^. During 2020, only 11 % of children aged 11–18 years and 24 % of adults aged 19–64 years achieved five portions of fruits and vegetables per day in the UK, and just 8 to 19 % of children and adults across age groups and sexes met government recommendations to consume no more than 5 % of daily energy intakes as free sugars^(4)^. Although there are many barriers to adherence to dietary guidelines, as reviewed elsewhere^(5)^, sleep habits are generally ignored as a contributing factor. However, there is accumulating evidence from observational studies and sleep deprivation intervention trials that sleep health is associated with diet quality and eating behaviours. Thus, diet is likely to be a significant mediating factor in the well-established links between inadequate sleep and increased risk of CVD, type 2 diabetes and obesity^(6–12)^.

Diagnosed sleep disorders such as sleep apnoea and insomnia are well-established risk factors for cardiometabolic diseases. However, sub-clinical sleep characteristics such as short sleep, irregular sleep and poor sleep quality are also important determinants of disease risk. Inadequate sleep duration is increasingly being recognised as a significant problem for the health of the nation, with well-described associations between poor sleep and impaired health^(12,13)^ and increased risk of accidents and injuries^(14)^. Very long sleep (≥ 9 h per night) is also associated with poorer health but those who habitually report these extended sleep durations are a diverse subpopulation that may have greater prevalence of morbidities that confound relationships with health^(12)^. Sleep timing is also emerging as a key predictor of risk of morbidity^(15)^, with later bedtimes, greater variability in sleep midpoint and irregular sleep durations being linked with increased biomarkers of cardiometabolic disease risk and adiposity^(16–19)^. The greater risk of impaired physical and mental health in long-term shift workers is attributable to chronic disruption to the physiological body clock in addition to shorter sleep duration and poor sleep quality. Social jetlag is a less severe form of chrono-disruption that affects approximately 16 % of the UK population^(20)^, manifesting as an earlier sleep midpoint during working days compared with free days (usually the weekend). It is also important to consider sleep quality, a term that encompasses frequency of awakenings, time taken to get to sleep (sleep latency) and time spent awake as a proportion of time spent in bed (sleep efficiency), measured both by subjective^(21)^ and objective^(22)^ assessment methodologies. This review will consider the duration, timing and quality of sleep as determinants of eating behaviour and food choices within the context of dietary interventions for weight management.

## Sleep duration and diet

The National Sleep Foundation recommends a sleep duration of 7–9 h per night for adults^(23)^. It is estimated that 6–10 % of adults suffer with diagnosed insomnia disorder^(24)^, but many more do not regularly achieve a healthy sleep duration simply due to work and lifestyle factors. In the UK, insufficient sleep is estimated to be the cause of 17 % of road deaths and injuries^(25)^, and simulation analysis estimates that the annual cost to the UK economy is around 2 % of GDP^(26)^. Accurate estimates of sleep duration are difficult to achieve at the population level as they are mainly derived from questionnaires asking about typical sleep duration in the week *v*. weekends, or work nights and free nights, or sometimes average sleep in 24 h including naps. Recent analysis by our group of the rolling cross-sectional National Diet and Nutrition Survey (NDNS), a representative sample of the UK population, across years 1–9 (2008/2009–2016/2017) suggests that prevalence of sleeping < 7 h (as an average across 7 d obtained by quesitonnaire) is 34 % in 19- to 64-year-olds^(20)^. In the USA, current estimates are consistent with the UK in that 36 % of adults aged 20 years or older sleep for 6 h or less on weekdays or workdays^(27)^. Long sleep is less prevalent; 7 % of the NDNS sample reported that they slept for more than 9 h on average^(20)^ and 7·5 % in the USA^(27)^.

Growing evidence from observational studies suggests that short sleep is associated with poor diet, including higher sugar intake^(27)^, and lower fibre^(28)^, fruit and vegetables^(29)^ and diet quality indices^(30,31)^. Our analysis of NDNS-RP data (2008/2009–2016/2017), based on 4-d food diaries from 5015 men and women aged 19–64 years, showed that short sleep (< 7 h) was associated with lower intake of fruits and vegetables, fibre, micronutrients (folate, Fe, vitamins B_12_, C and K), protein and higher intake of non-milk extrinsic sugars as well as a higher carbohydrate:non-starch polysaccharide fibre ratio (adjusted for age, sex, BMI, total energy intake, ethnicity, economic status, smoking status, alcohol intake, number of children below 4 years of age and long-standing illness)^(20)^. Short sleep has also been associated with more snacking and eating out, less variety in the diet, a greater likelihood of missing breakfast and consuming fewer main meals, increasing intake of energy in the evening and irregular eating patterns, as reviewed by Dashti *et al.* ^(32)^.

The complexity of eating behaviour is such that we cannot assume that the observational relationships between sleep duration and diet are causal in nature. Furthermore, it is difficult to gauge the directionality of potential causal relationships without intervention studies. Multiple randomised controlled trials have investigated the impact of short-term sleep restriction on energy intake (mostly under highly controlled conditions in a laboratory setting), overall showing that short sleep duration (3 h 45 min to 5 h 30 min sleep per night) results in increased energy intake of 385 kcal per day^(33)^. In most cases, these studies investigated acute responses to sleep regimes lasting 1–7 d; therefore, these findings may not be translatable to free-living humans.

A more realistic experimental setting would be to increase sleep duration in free-living short-sleepers, but this requires an individual to make significant behaviour changes alongside their usual working/home life and few studies with this design exist. Our group conducted an randomised controlled trial (the SLuMBER study) in healthy adults to explore the feasibility of extending sleep duration in free-living people and to pilot the effects of a sleep extension intervention on diet and cardiometabolic health markers^(34)^. The SLuMBER Study was a parallel 2-arm, 4-week sleep extension behaviour intervention study in forty-three young, healthy short-sleepers (82 % female), where the intervention group received personalised sleep hygiene counselling with a focus on changing ∼4 key behaviours that prevented them sleeping for 7 h or more (e.g. having a dark, uncluttered bedroom, avoiding electronic screens late in the evening, keeping a regular bedtime, not exercising too close to bedtime, avoiding large meals, caffeine and alcohol close to bedtime, meditation for stress, etc)^(34)^. Following the sleep extension intervention, 86 % of the sleep extension group increased time in bed and 50 % increased actual sleep duration (excluding awakenings), measured objectively by wrist actigraphy. Interestingly, there was a decline in sleep quality in the intervention group relative to the control group; this meant that actual time spent asleep (minus awakenings) increased by a modest 32 min despite average sleep episodes increasing by 52 min in the sleep extension group relative to the control group. This underlines the importance of conducting longer term studies to understand whether adaptation to new sleep habits can occur leading to improvements in sleep quality over time and to evaluate the long-term durability of sleep behaviour change.

Participants received no dietary advice as part of the SLuMBER study intervention (other than that relating to caffeine, alcohol and not eating close to bedtime), but analysis of 7-d food diaries showed that there was a significant 10 g/d decrease in free sugars intake in the sleep extension group relative to the control group^(34)^. These findings align with the observational data and suggest that cross-sectional associations between short sleep and higher sugar intake is at least in part attributable to a direct effect of short sleep on consumption of foods high in sugar^(34)^. Similarly, Tasali *et al*.^(35)^ conducted a parallel 2-arm, 2-week randomised controlled trial in eighty healthy young adults (49 % female) with overweight and reporting sleep < 6·5 h/night; the intervention group receiving personalised sleep hygiene counselling resulted in an increase in sleep duration of 1·2 h per night in the sleep extension group (measured by wrist actigraphy), with no differences in sleep efficiency observed. Again, no dietary advice was provided, but nevertheless energy intake, calculated from changes in body composition and energy expenditure (doubly labelled water), decreased in the sleep extension group by 270 kcal/d relative to the control group, and an inverse correlation between energy intake and sleep duration changes was reported^(35)^. These results further support the direct effect of sufficient sleep duration on decreased intake of energy-dense foods.

## Sleep quality and diet

Whilst consideration of sleep duration is essential in evaluating an individual’s overall health, it does not capture the whole picture in terms of optimal sleep function. Sleep quality is equally important, particularly as there is a U-shaped relationship between sleep duration and sleep quality, with poorer sleep quality being more prevalent in both short sleepers and long sleepers (> 9 h)^(36)^. Sleep quality is often evaluated subjectively both clinically and in research using the Pittsburg Sleep Quality Index^(37)^, which asks a series of questions to generate component scores for overall sleep quality, latency, duration, efficiency, disturbances, use of sleeping medications and daytime sleepiness and an overall Pittsburg Sleep Quality Index score for perceived typical sleep quality. Relatively few nutritional/weight management studies have used objective methods, for example by actigraphy, to assess measures of sleep quality during defined time periods, including frequency and duration of awakenings/sleep fragmentation, wake time after sleep onset, sleep latency and sleep efficiency. Unlike sleep duration. there are no national/international guidelines for sleep quality, but a general recommended cut-off is a sleep efficiency of 85 %; in other words, total sleep time as a percentage of time in bed from the point of intention to sleep (including sleep latency, total sleep time, time awake after initial sleep onset and time after final awakening attempting to get back to sleep) should ideally be more than 85 %^(38)^. Sleep efficiency declines steadily with age^(36)^, and it is approximated that 25 % of 41 to 65-year-olds in the Netherlands, UK and USA were not reaching a sleep efficiency estimate of 85 %^(36)^.

Observational studies from various countries have reported associations between better sleep quality and adherence to the Mediterranean diet, higher intake of fruits and vegetables and lower dietary inflammatory index and high sugar/refined starch food intakes, as reviewed by Godos et al.^(39)^. Moreover, poorer sleep quality was associated with a greater weight of food intake, higher intake of added sugars and caffeine, lower dairy intakes and lower unsaturated fat, and longer sleep onset latency was associated with higher energy intakes in 495 USA women aged 20–79 years^(40)^.

Both poor sleep quality and poor diet quality are end products of psychological factors such as work or family stress, anxiety or loneliness leading to emotional distress^(41)^; therefore, it is important to determine whether poor sleep quality itself can initiate or exacerbate unhealthy food choices to better support the design of intervention strategies to improve diet. Trials are lacking that include an intervention arm specifically aiming to improve sleep quality (by non-pharmacological means, e.g. sleep hygiene, cognitive behavioural therapy) with diet as an outcome variable. However, there is strong evidence that improving sleep quality can positively impact mental health that is likely to enable healthier dietary behaviours^(42)^. In addressing the reverse relationship – the effect of diet on sleep quality – there is more evidence available. Macronutrient composition may influence sleep quality. For example, consumption of a higher proportion of energy as protein during energy-restricted diets improved Pittsburg Sleep Quality Index scores compared with lower relative protein intakes^(43)^. Another small crossover (*n* 12) study in young males reporting that consuming higher glycaemic index (GI) rice in the evening may reduce sleep latency compared with lower GI rice^(44)^, further supported by a subsequent small crossover study (*n* 10) confirming that higher-GI evening meals reduced sleep latency and total wake time, and also increased sleep efficiency in recreational trained male athletes^(45)^. These provide preliminary evidence that eating high GI starchy foods before bedtime promote better sleep quality, but interventional evidence is lacking on the impact of whole dietary patterns on markers of sleep quality and currently the only data available are derived from observational studies that show associations between dietary patterns such as the Mediterranean diet and sleep quality, as reviewed by Zuraikat et al.^(46)^

## Sleep timing and diet

Inconsistency in sleep timings and irregular sleep duration may also be a determinant of poor diet quality. The greater risks of cardiometabolic diseases^(47,48)^, obesity^(49)^ and metabolic dysfunction^(50)^ from working shifts (currently estimated at 14 % of the working population in the UK^(51)^) are well documented. Although there is a direct effect of circadian misalignment on cardiometabolic health^(52)^, it is also likely that part of the increased cardiometabolic disease risk is attributable to the unfavourable influence of undertaking shift work on diet quality^(53,54)^. A roughly similar proportion of the population do not work night shifts yet are exposed to a milder form of circadian misalignment directly related to working schedules and in many case the associated commuting time. The requirement to wake early to travel to work or place of education for an 08.00–09.00 start can lead to marked differences in sleep/wake times between workdays and free days. This discordance between sleep onset and waking times across the work/non-work parts of the week is known as ‘social jetlag’, calculated as the different in sleep-midpoint between work days and non-work days^(55)^. Estimates from the UK NDNS survey show that around 14 % of adults aged 19–64 years have a > 2 h difference in sleep duration between week days and weekend days^(20)^, and 46 % of respondents to the Munich ChronoType Questionnaire (*n* 185 333) had a delay in midsleep on free days compared with workdays of more than 1 h (17 % more than 2 h) in 2017^(56)^.

The impact of shift work on eating habits has been investigated in many countries and cultural settings and, although there are some inconsistencies, evidence to date suggests that adverse dietary behaviours are a critical risk factor for the health of night shift workers. A systematic review of thirty-three observational studies found more meal skipping and nocturnal eating in night and rotating shift workers, as well as increased consumption of soft drinks, higher energy and saturated fat intakes and lower fibre intake^(57)^. Social jetlag has also been associated with poor diet in observational studies^(58,59)^. Analysis of Y1-4 NDNS data by Almoosawi and colleagues showed that both negative and positive differences in sleep duration (sleep midpoints not being available in this data set) on week nights and weekend nights were associated with reduced diet quality^(60)^. Our study of a larger NDNS data set (Y1^–^9) has shown that social jetlag is associated with higher non-milk extrinsic sugar intakes in UK adults aged 19–64 years (comprehensively adjusted), although there were no differences in fibre or fruit and vegetable intakes^(20)^. When investigating proportions of consumers of different foods, more individuals with social jetlag consumed less healthy snack foods such as chocolate and crisps and fewer consumed ‘fruit’ and ‘oily fish’. Upon stratification of the sample for short and adequate sleep duration, social jetlag status was not associated with any further reduction in fibre intake in short sleepers, but in adequate sleepers (7–< 9 h) those with social jetlag had lower fibre intakes^(20)^. Furthermore, He *et al.* reported that sleep variability in 324 USA adolescents (the sd of 7-day sleep duration) was associated with higher energy intakes, whereas there were no associations with sleep duration^(61)^. These findings suggest that mild circadian misalignment may also influence dietary behaviour independently of sleep duration.

There is a lack of evidence from randomised controlled trials on the impact of manipulating sleep timing (whilst keeping sleep duration constant) on diet quality or eating behaviour, although there are a handful of dietary interventions targeted at night shift workers to improve health outcomes^(62)^. The scarcity of intervention studies on the impact of sleep timing on dietary behaviours is most likely due to the challenges of designing this type of behavioural intervention in a free-living study setting. One small 2 × 2 crossover study (*n* 5) in a controlled laboratory setting imposed a schedule of normal (midnight-08.00) and late (03.30–11.30) sleeping in addition to normal and delayed eating, with each intervention phase lasting 5 d separated by a 28 d wash-out. The results showed that late sleep timing did not increase energy intake, saturated fat or sugar intakes, possibly because the strict regime outside of participants’ normal environment inhibited natural behaviours^(63)^.

## How do sleep habits influence dietary intakes?

**Fig. 1.**
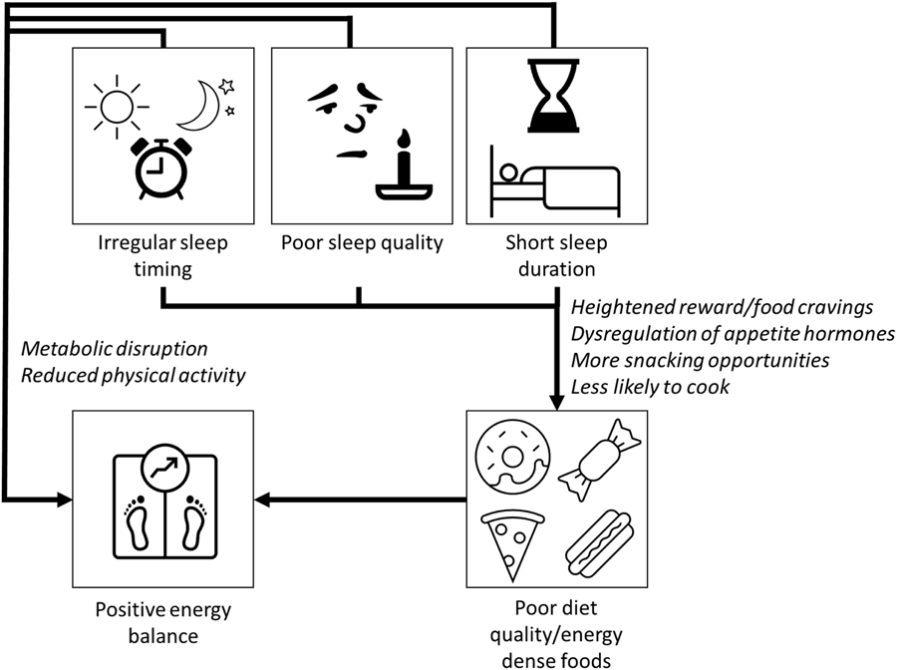
Short sleep duration, poor sleep quality and irregular sleep timing have adverse effects on several biological processes that may directly increase risk of obesity and cardiometabolic diseases. Inadequate sleep can increase the activity of the sympathetic nervous system, promote inflammation and oxidative stress and lead to disturbances in lipid metabolism, which then can accelerate the progression of type 2 diabetes and CVD. Short sleep may also cause weight gain through changes to metabolism, reduction in physical activity and by increased energy intake. Sleep deprivation causes food cravings for sugary/fatty foods due to changes in reward processing in the brain and a heightened sense of taste. Dysregulation of appetite pathways (increased ghrelin and reduced leptin production) will increase hunger and decrease satiety. Going to bed late or working night shifts increases likelihood of snacking, and sleep deprivation and irregular sleep episodes are associated with reduced likelihood of cooking at home.

Short sleep may cause changes in the brain that lead to a more intense sense of taste^(64)^ and a greater desire for highly palatable sugary/fatty foods via the neuronal reward network^(65,66)^. One night’s modest sleep deprivation (with midpoint of sleep episode held constant to control for circadian effects) led to increases in hunger, increased food cravings, increased susceptibility to food reward and selection of larger portions at lunchtime in a randomised controlled trial in twenty-four young healthy females compared with a full night’s sleep^(67)^. Another explanation is dysregulation of appetite hormones such as ghrelin and leptin^(68–70)^, although evidence is somewhat inconsistent^(71)^. In determining the effects of sleep timing on appetite and reward behavior, it is difficult to disentangle circadian effects from the effects of sleep curtailment, as night shift work is associated with short sleep duration and reduced sleep quality^(72)^, and social jetlag is also more common in short-sleepers^(20)^. However, observational data indicate that social jetlag is independently associated with increased appetite for food^(73)^ and preliminary evidence suggests independent effects of social jetlag on brain regions associated with reward and food craving^(74)^. In most cases, short sleep, poor sleep quality and irregular sleep timing coincide, and it is likely that they all contribute in multiple ways to poor diet quality and increased risk of weight gain (Fig. 1).

## Sleep advice as an adjunct to weight management programmes

The previous sections have shown that sleep duration, quality and timing are all likely to significantly influence an individual’s eating behaviour and food choices throughout the day. This presents an opportunity to pair sleep health guidelines with weight management programmes that focus on diet and exercise to improve compliance and weight loss. Short sleep duration is associated with obesity^(6,75)^ and increased abdominal fat^(76)^, and short-term (4-d) sleep restriction induces a small net increase in body weight^(77)^. Although relationships between body fat and sleep are believed to be bidirectional, healthy sleep habits are likely to facilitate healthier dietary choices and reduced food cravings; for example, better sleep health at baseline was associated with greater weight loss in adults with overweight/obesity undergoing a 1 year weight management programme^(78)^. Currently, studies on efficacy of diet and/or exercise plus sleep interventions (extending sleep durations, improving sleep quality or standardising sleep timings) are scarce. Therefore, an evidence base to justify the introduction of sleep advice as an integral component of weight management programmes is lacking. One study randomised forty-six patients with overweight/obesity to either a weight loss (diet, exercise and cognitive behavioural therapy) plus sleep intervention arm, or weight loss alone and showed there was a greater amount of weight loss after 12 weeks in the sleep group, although drop-out rates were high and sleep was not monitored objectively, thus more evidence is needed^(79)^. Another study in fifty-two adolescents with obesity demonstrated a greater degree of weight loss and reduction in waist circumference following a 4-week energy-restricted diet with sleep twenty-five participants with overweight/obesity to either a 4-week baseline sleep health intervention or a control education programme before they embarked on the same weight loss intervention, but there was poor compliance to sleep extension and no difference in weight loss at the end of the 18 week weight loss intervention, indicating that continued sleep health support may needed throughout weight management programmes for efficacy^(81)^. Finally, Wang *et al.* (*n* 36) combined an 8-week weight loss intervention with 1 h per night, 5 nights per week sleep restriction (net weekly sleep deficit 169 min), which resulted in similar weight loss to the weight loss control group, but reduced fat mass loss^(82)^. Only a very limited number of weight management service providers or health organisations currently provide general sleep advice or sleep hygiene guidelines as part of their guidelines for weight management (author’s unpublished data). Clearly, there is an urgent need for longer term interventions to improve sleep health in tandem with weight management and weight loss maintenance programmes to provide evidence needed for incorporation into national clinical guidelines and wider adoption by weight loss providers.

## Concluding remarks

The interaction between sleep, diet and health has been in a state of flux in the last couple of years due to sudden changes in working practices during the COVID-19 pandemic. For many people, this has resulted in the adoption of hybrid working patterns that could potentially reduce the degree of social jetlag^(83)^, although the overall impact on sleep health and well-being of hybrid working relative to 100 % office working or fully working at home is not yet clear^(84)^. More research is needed to understand the potential impact of increased working from home on sleep habits, dietary behaviours and risk of weight gain so that employers have all the information they need to provide support for workers’ health and well-being. Personalisation of dietary advice in weight loss interventions may be even more impactful if personalisation of sleep hygiene advice is delivered in tandem^(85)^, and developing ways to deliver personalised combination lifestyle interventions at scale should be a priority for governments and health authorities. Life stage (adolescence, university, parenting, menopause and retirement), biological age and sex should all be taken into consideration as sleep requirements, sleep quality and sleep timing and the physiological, psychological and dietary consequences of poor sleep health all vary across these characteristics^(86)^. In conclusion, poor sleep habits affect a significant proportion of the UK population, and targeting ways to improve the sleep health of the nation may prove to be an essential component of the overall strategy to improve diets and prevent obesity and chronic diseases.
